# Proceedings of the Mississippi State Dental Association

**Published:** 1881-08

**Authors:** 


### ARTICLE II.

### *Proceedings of the Mississippi State Dental Association.*

The Sixth Annual Convention of the Mississippi State Dental Association was held in the rooms of the Glee Club, at Jackson, Miss., on the 17th and 18th of May, 1881.

The attendance was unusually large, and by the activity and interest of the members of the association, evinced the rapidity of the strides by which it has advanced in popular and professional favor.

Comparatively a young organization, the strength and quality of its membership will go far to elevate and sustain in the minds of the people the character of the profession of which it is the representative. The necessity of membership as an evidence of high standing and attainments is making itself felt throughout the ranks of the profession, and the day is not far distant when with the aid of intelligent legislation, the strongest plea for which is our own united effort—freed of *impediments*, we can advance rapidly in the field of research and improvement.

Unfavorable as it may seem to some, the omissions of the usual series of essays and the consequent discussions which have been so prolific a source of information, was not indicative of any lack of interest or want of industry on the part of members, but shows rather, considering the nature of the work accomplished, the importance and necessity of that very work itself, the object of which is to secure results of a nature which will leave us free to advance with greater confidence and success in whatever direction duty or science may lead.

The meeting was called to order at 10 o'clock a. m., on Tuesday, the 17th, Dr. R. J. Miller, President, in the chair. The Secretary being absent, Dr. Geo. W. Rembert was elected Secretary pro tem.

The roll being called, upon motion of Dr. Geo. W. Rembert, the regular order of business was suspended and a vote was taken resulting in the election of the following named applicants for active membership, then in waiting in the ante-room, namely:

Dr. E. E. Spinks, Meridan.

Dr. Joseph B. Pleasants, Aberdeen.

Dr. B. S. Byrnes, Port Gibson.

Dr. A. A. Dillehay, Meridan.

Dr. J. A. Watson, Lexington.

Dr. A. S. Oliver, Moscow.

Regular order of business resumed, and proceedings of last regular meeting read and approved.

The President's annual address, being next in order, was delivered in an able and impressive manner, containing many suggestions of the highest value, both to the popular and professional mind.

Upon motion of Dr. A. H. Hilzheim, it was resolved that the President's address be received, filed with, and published as a part of the proceedings of this meeting.

[Owing to its voluminous nature the Secretary deemed its publication at present impracticable.]

On motion of Dr. J. D. Miles, the Association was adjourned until 3:30 p. m.

---

Afternoon session, 3:30 p. m. Meeting called to order by the President.

Report from Treasurer showed an exceedingly healthful condition of the finances, the Association being clear of debt and with a good balance in the Treasury.

Committee on Arrangements reported that they had secured a liberal reduction of fare to members on the railroads in the State to which they applied except the Mississippi & Tennessee R. R., and reduced rates at both the Spengler House and the Edwards House. Report received and committee discharged.

Committee on publication of annual proceedings, membership certificates, notice cards, &c., made their report, which was received, ordered filed, and committee discharged.

Committee appointed at previous meeting to prepare for publication a treatise, educating the public mind on dental subjects, reported progress, and asked for further time. On motion of Dr. Joseph B. Pleasants, committee allowed until next regular meeting for final report.

The Executive Committee recommended for active membership the following absentees: Dr. R. C. Jeffries, Natchez, Dr. Isham B. Rembert, Fayette, and Dr. E. B. Robbins, Vicksburg. A vote was then taken, resulting in their unanimous election.

The annual election of officers for the ensuing term resulted as follows:

President—Dr. J. D. Miles, of Vicksburg.

1st Vice-President—Dr. E. E. Spinks, Meridan.

2d Vice-President—Dr. Joseph B. Pleasants, Aberdeen.

Secretary and Treasurer—Dr. Geo. W. Rembert, Natchez.

By vote of the Association the President-elect was invited to take the presiding officer's chair.

Dr. Geo. W. Rembert moved that the thanks of the Association be tendered Dr. R. J. Miller, retiring President, for the very able and efficient services tendered to this Association while its presiding officer. Motion unanimously adopted.

Dr. A. A. Dillehay moved the Association adjourn to meet in evening session, at 8 p. m., at the parlors of the Edwards House.

---

Evening session, 8 p. m., May 17th. Society called to order, President Dr. J. D. Miles presiding.

The President stated that he would withhold the appointment of the standing committees until the final session. Under the head of new business the question of locating the Association at Jackson, its expediency, &c., was introduced and elicited free discussion by the members.

Dr. Geo. W. Rembert offered the following resolution: Resolved, That the next annual meeting of this Association be held at Jackson, Miss., on the 3d Tuesday in January next, A. D. 1882, and that each annual meeting thereafter be held at Jackson at such time as this Association may direct. Motion carried.

At the suggestion of Dr. J. D. Miles, and on motion of Dr. E. E. Spinks, it was

Resolved, 1st, That Art. 4th, Sec. 2d of the constitution be changed to read as follows: "And pay to the Treasurer the sum of $3 as initiation fee."

2d, That Art. 1st. Sec. 1st of by-laws be changed to read as follows: "The annual dues of this Association shall be one dollar, payable in advance, at each regular meeting."

On motion of Dr. A. A. Dillehay, it was

Resolved, That each member of this Association who may be in arrears for annual dues, shall be entitled to a clear receipt from the Treasurer upon payment into the Treasury of this Association the sum of one dollar for each year of his membership, less the amount previously paid.

Dr. A. H. Hilzheim offered the following:

Resolved, That it shall be the duty of the incoming President, at each regular meeting, to appoint committees of three members to prepare essays, on any subject connected with dentistry, to be read before the next annual meeting, and that this resolution shall operate as an amendment to that portion of Art. 5th, Sec. 5th of the by-laws referring to appointment by the Executive Committee of committees to prepare essays. Carried.

Dr. Geo W. Rembert submitted the following resolution, which was carried:

Resolved, That a committee of three be appointed by the President, to draft a Code of Ethics and Fee Bill for this Association, and present for adoption before final adjournment of this Convention.

Committee—Drs. Geo. W. Rembert, E. E. Spinks and A. H. Hilzheim.

On motion of Dr. B. S. Byrnes, it was

Resolved, That a committee of three be appointed to revise the Constitution and By-Laws, embody therein the

Code of Ethics and Fee Bill, and present at next annual meeting.

Committee—Drs. B. S. Byrnes, A. H. Hilzheim and Joseph B. Pleasants. On motion of Dr. Dillehay the Secretary was added.

Dr. Ben. Jones offered the following resolution:

Resolved, That this Association, recognizing the worth of Dr. J. H. Andrews, of Lexington, Miss., do elect him an honorary member of this Association. Unanimously adopted.

Dr. A. S. Oliver moved the Association be adjourned, to meet at the Glee Club rooms at 8 o'clock, a. m. Motion carried.

---

MORNING SESSION OF SECOND DAY.

Association called to order at 8 o'clock a. m., President Miles in the chair.

Dr. J. D. Miles, who was appointed by the American Dental Association to aid in compiling a directory of all dentists in the United States, submitted to this Association for correction, a printed list of dentists in Mississippi, which was read by the Secretary, and certain corrections then made. Being still incomplete, the further assistance of the Association was requested in perfecting the same.

Dr. Geo. W. Rembert moved that a committee of three be appointed by the President, whose duty it shall be to compile into pamphlet form all recent State dental legislation in the United States, with a suitable preface, setting forth therein the objects of, and good to be attained by, the enactment of such a law as the bill which is to be thereunto appended; and the said committee be authorized to have published 300 copies of said pamphlet, and that one copy of the same be mailed to each member of the State Legislature, with the compliments of this Association. Motion carried.

Committee—Drs. Geo. W. Rembert, A. A. Dillehay and R. C. Jeffries. On motion of Dr. A. H. Hilzheim, the President was added.

After brief intermission the committee on Code of Ethics and Fee Bill reported. Report received, Code of Ethics and Fee Bill adopted, and committee discharged.

---

### CODE OF ETHICS.

### ARTICLE I.

*The Duties of the Profession to their Patients.*

Sec. 1. The dentist should ever be ready to respond to the wants of his patrons, and should fully recognize the obligations involved in the discharge of his duties toward them. As they are, in most cases, unable to correctly estimate the character of his operations, his own sense of right must guarantee faithfulness in their performance. His manner should be firm, yet kind and sympathizing, so as to gain the respect and confidence of his patients, and even the simplest case committed to his care, should receive that attention which is due to operations performed upon living sensitive tissue.

Sec. 2. It is not expected that the patient will possess a very extended, or a very accurate knowledge of professional matters. The dentist should make due allowance for this, patiently explaining many things that may appear quite clear to himself, thus endeavoring to educate the public mind, so that it will properly appreciate the beneficent efforts of our profession. He should encourage no false hopes by promising success, when in the nature of the case there is uncertainty.

Sec. 3. The dentist should be temperate in all things, keeping both mind and body in the best possible health, that his patients may have the benefit of that clearness of judgment and skill, which is their right.

### ARTICLE II.

*Maintaining Professional Character.*

Sec. 1. A member of the Dental profession is bound to maintain its honor, and labor earnestly to extend its sphere of usefulness. He should avoid everything in language and conduct, calculated to dishonor his profession, and should ever manifest a due respect for his brethren.

Sec. 2. The person and office arrangements of the Dentist should indicate that he is a gentleman; and he should sustain a high-toned moral character.

Sec. 3. It is unprofessional to resort to public advertisements calling attention to peculiar styles of work, lowness of prices, special modes of operating; or to claim superiority over neighboring practitioners; to go from house to house to solicit operations, except in country practices; to recommend or circulate patent nostrums; or to perform any other similar acts.

Sec. 4. When consulted by the patient of another practitioner, the Dentist should guard against inquiries or hints disparaging to the family Dentist, or calculated to weaken the patient's confidence in him. Nor should he by misrepresentations, or by offering other inducements, endeavor to win the patronage of the patient from his brother Dentist.

Sec. 5. When general rules shall have been adopted by members of the profession practicing in the same localities, in relation to fees, it is unprofessional and *dishonorable* to depart from those rules, except in charity cases, and only then when the patient is willing to accept it as charity; provided, this shall not apply to clergymen and physicians, and their families. And it is ever to be regarded as unprofessional to warrant operations or work, as an inducement to patronage.

### ARTICLE III.

*The Relative Duties of Dentists and Physicians.*

Dental Surgery is a specialty in medical science. Physicians and Dentists should both bear this in mind. The Dentist is professionally limited to diseases of the dental organs and the mouth. With these he should be more familiar than the general practitioner is expected to be; and while he recognizes the superiority of the physician in regard to the diseases of the general system, the latter is under equal obligations to respect his higher attainments in his specialty. When this principle governs, there can be no conflict, or even diversity of professional interests.

ARTICLE IV.

*The Mutual Duties of the Profession and the Public.*

Dentists are frequent witnesses, and at the same time the best judges, of the impositions perpetrated by quacks; and it is their duty to enlighten and warn the public in regard to them. For this, and the many benefits conferred by the competent and honorable Dentist, the profession is entitled to the confidence and respect of the public, who should always discriminate in favor of the true man of science and integrity, against the empiric and impostor.

(The Secretary deemed the publication of the Fee Bill inexpedient.)

Delegates elected to the Southern Dental Association, which convenes at Ashville, North Carolina, on the last Tuesday in July, 1881—Drs. Jos. B. Pleasants, and Geo. W. Rembert.

Delegates elected to the American Dental Convention—Drs. A. Riser and A. H. Hilzheim.

Delegates to the American Dental Association—Drs. W. H. Marshall and A. A. Dillehay.

The following committees were appointed by the President.

Committee on Arrangements—Drs. Isham B. Rembert, E. B. Robbins and C. C. Marshall.

Executive Committee—Drs. B. S. Byrnes, A. S. Oliver and J. A. Watson.

Committee on Physiology and Dental Histology—Drs. W. T. Martin, A. A. Dillehay, and W. H. Marshall.

Committee on Dental Pathology and Therapeutics—Drs. Joseph B. Pleasants, M. C. Marshall and B. S. Byrnes.

Committee on Operative Dentistry—Drs. Geo. W. Rembert, J. B. Askew and A. H. Hilzheim.

Committee on Mechanical Dentistry—Drs. A. Riser, Ben Jones and A. S. Oliver.

Committee on Dental Education and Literature—Drs. I. O. Payne, R. J. Miller and Isham B. Rembert.

Committee on Dental Appliances—Drs. E. E. Spinks, R. C. Jeffries and J. A. Watson.

On motion of Dr. Geo. W. Rembert, the President was added to the "Committee on Dental Education and Literature."

On motion of Dr. R. J. Miller it was

Resolved, That a vote of thanks of the Mississippi State Dental Association be extended to the Edwards House ; also to the Spengler House at Jackson, Miss., for reduced rates to members.

On motion of Dr. E. E. Spinks, it was

Resolved, 1st. That the thanks of this Association be voted to the following railroads for courtesies extended in reduced rates to members, to-wit: N. O., St. L. & C. R. R., V. & M. R. R., Mobile & Ohio R. R., and Memphis & Charleston R. R.

Resolved, That the thanks of this Association be tendered the Glee Club, of Jackson, Miss., for the courtesies extended us in the use of their rooms.

On motion of Dr. Geo. W. Rembert, it was

Resolved, That the thanks of this Association be extended the Natchez *Democrat* and the Natchez *New Era* for the gratuitous publication of notices of this meeting.

Dr. A. H. Hilzheim moved a vote of thanks to the Jackson *Clarion* and *Comet* for gratuitous notices. Carried.

Dr. J. D. Miles suggested a vote of thanks from this Association to the Vicksburg *Herald* and Vicksburg *Commercial*, which, upon motion, was extended.

There appearing no further business the Association adjourned to meet at Jackson, Miss., on 3d Tuesday in January, 1882.

